# Climate variation during the Holocene influenced the skeletal properties of *Chamelea gallina* shells in the North Adriatic Sea (Italy)

**DOI:** 10.1371/journal.pone.0247590

**Published:** 2021-03-04

**Authors:** Alessandro Cheli, Arianna Mancuso, Michele Azzarone, Simona Fermani, Jaap Kaandorp, Frederic Marin, Devis Montroni, Iryna Polishchuk, Fiorella Prada, Marco Stagioni, Giovanni Valdré, Boaz Pokroy, Giuseppe Falini, Stefano Goffredo, Daniele Scarponi

**Affiliations:** 1 Marine Science Group, Department of Biological, Geological and Environmental Sciences, University of Bologna, Bologna, Italy; 2 Fano Marine Center, The Inter-Institute Center for Research on Marine Biodiversity, Resources and Biotechnologies, Fano, Italy; 3 Department of Biological, Geological and Environmental Sciences, University of Bologna, Bologna, Italy; 4 Department of Chemistry ‘Giacomo Ciamician’, University of Bologna, Bologna, Italy; 5 Computational Science Laboratory, Faculty of Science, University of Amsterdam, Amsterdam, The Netherlands; 6 UMR CNRS 6282 Biogéosciences, Université de Bourgogne—Franche-Comté, Dijon, France; 7 Department of Materials Sciences and Engineering and the Russell Berrie Nanotechnology Institute, Technion–Israel Institute of Technology, Technion City, Haifa, Israel; 8 Marine Biology and Fisheries Laboratory of Fano, Department of Biological, Geological and Environmental Sciences, University of Bologna, Italy; University of Pisa, ITALY

## Abstract

Understanding how marine taxa will respond to near-future climate changes is one of the main challenges for management of coastal ecosystem services. Ecological studies that investigate relationships between the environment and shell properties of commercially important marine species are commonly restricted to latitudinal gradients or small-scale laboratory experiments. This paper aimed to explore the variations in shell features and growth of the edible bivalve *Chamelea gallina* from the Holocene sedimentary succession to present-day thanatocoenosis of the Po Plain-Adriatic Sea system (Italy). Comparing the Holocene sub-fossil record to modern thanatocoenoses allowed obtaining an insight of shell variations dynamics on a millennial temporal scale. Five shoreface-related assemblages rich in *C*. *gallina* were considered: two from the Middle Holocene, when regional sea surface temperatures were higher than today, representing a possible analogue for the near-future global warming, one from the Late Holocene and two from the present-day. We investigated shell biometry and skeletal properties in relation to the valve length of *C*. *gallina*. Juveniles were found to be more porous than adults in all horizons. This suggested that *C*. *gallina* promoted an accelerated shell accretion with a higher porosity and lower density at the expense of mechanically fragile shells. A positive correlation between sea surface temperature and both micro-density and bulk density were found, with modern specimens being less dense, likely due to lower aragonite saturation state at lower temperature, which could ultimately increase the energetic costs of shell formation. Since no variation was observed in shell CaCO_3_ polymorphism (100% aragonite) or in compositional parameters among the analyzed horizons, the observed dynamics in skeletal parameters are likely not driven by a diagenetic recrystallization of the shell mineral phase. This study contributes to understand the response of *C*. *gallina* to climate-driven environmental shifts and offers insights for assessing anthropogenic impacts on this economic relevant species.

## Introduction

Evaluating how marine ecosystems could respond to near-future global warming is critical to design proper conservation and management strategies, especially in coastal areas with increasing urbanization and resource overexploitation.

In the marine realm, calcifying macroinvertebrates such as corals, brachiopods and mollusks produce hard structures for support and protection that constitute high-resolution archives recording the environmental conditions that have prevailed during their life [1, 2]. Through the control exerted by intraskeletal macromolecules, mollusks can exert imprints on calcium carbonate biomineralization [3], influencing the polymorphism, morphology and chemistry of the shell in response to environmental changes [4–6]. Those biogenic structures can be useful tools to reconstruct the historical effects of climate change on marine organisms, thus allowing a better understanding of near-future dynamics.

Quantifying the effect of near future climate change on marine calcifying organisms requires long-term multi-generational studies for assessing their adaptability to changing environmental conditions [7]. Nevertheless, such studies are difficult to address in laboratory conditions. Natural latitudinal gradients could represent an alternative to laboratory experimental studies. In fact, this methodology allows to evaluate the effects of different environmental conditions, like temperature variations, along large-scale spatial gradients [7, 8]. A complementary approach is to investigate the recent fossil record. This line of research gives access to an archive of ecological responses to past climate transitions that could elucidate near-future scenarios of marine ecosystems under global warming [9, 10].

During the Holocene some time intervals were warmer than the present. Of these warmer periods, the longest was from about 9,000 to about 5,000 years before present (BP) (*i*.*e*., Holocene climate optimum HCO), with significantly higher temperatures than today at high latitudes (up to 4°C [11]). Holocene sedimentary successions are characterized by well-preserved remains of mollusk taxa with well-known ecological needs. Thus, it preserves a centennial record of environmental and biological dynamics that lead to present-day ecosystems. In this context, the recent sedimentary succession of the Po Plain-Adriatic Sea system (Italy) has been extensively investigated in the last decades and offers a high-resolution stratigraphic framework (for details see S1 Text “Geological setting” in S1 File; [12–21]). Hence, biomineralization dynamics in relation to millennial scale climate change can here be investigated in a well-resolved climate and stratigraphic framework.

Among economically relevant mollusks of the Adriatic Sea, the infaunal bivalve *Chamelea gallina* seems to be particularly sensitive to environmental changes, showing shell morphology variations in response to environmental change [22–25]. Previous studies have mainly focused on population dynamics, shell growth and composition of this species in the present-day Mediterranean and along latitudinal gradients [24, 25]. In contrast, there is no information about shell variations in relation to climate-driven environmental change along temporal gradients.

This study aimed to investigate the variations in skeletal features of *C*. *gallina* assemblages during the last 8000 years from shoreface deposits and active shoreface settings of the Po-Adriatic system (Italy). This allowed to assess phenotypic variation occurred in time with different environmental conditions and determine how the impact of anthropogenic warming could affect this economically important bivalve species in the future. Biometry, composition and crystal structure of *C*. *gallina* shell were investigated in five shoreface-related horizons: two from the Middle Holocene, one from the Late Holocene and two from modern thanatocoenoses. Since diagenetic processes can occur over time, analyses of the taphonomic degradation status of the sub-fossil shells were carried out before comparing the results with modern thanatocoenosis.

## Materials and methods

### Specimen collection

**Fig 1:**
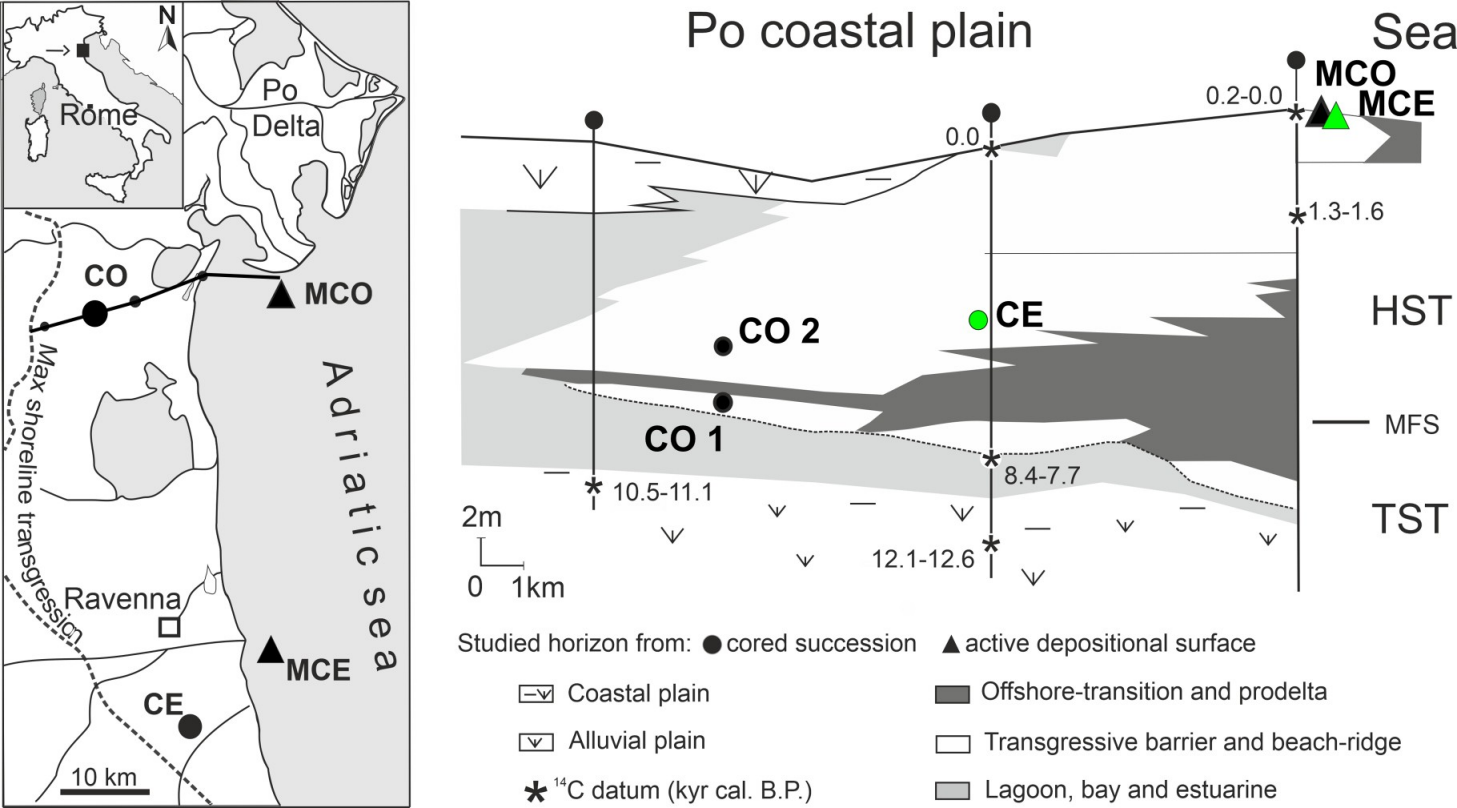
Study area map and stratigraphic framework of the latest Quaternary Po coastal plain-sedimentary succession (reprinted slightly modified from Scarponi *et al*. [31], under a CC BY license, with permission from the geological society of America original copyright 2013). In the left panel, black dots mark the locations of sampled cores (CO, CE), black triangles mark sampled thanatocoenosis from present-day shoreface environments (MCE, MCO), and the black solid line represents the along-dip cross section of the Po coastal plain (right panel). Right panel shows sketched climate driven environmental changes within the study area during the Holocene, see also Fig 5; the dashed line indicates the wave ravinement surface (see also S1 Text in S1 File) and green-filled symbols represent projected samples on the cross-section. Acronyms: MFS = maximum flooding surface; TST = transgressive systems tract; HST = highstand systems tract.

This study has been conducted on remains of *C*. *gallina* from areas not privately owned or protected. The species collected in this study is not protected or endangered. No specific permits were required to collect shell material for scientific research from sediment cores or targeted areas. Sub-fossil specimens (Holocene in age) of *C*. *gallina* were sampled from sediment cores of the Po Coastal Plain (Italy) and drilled as part of a multidisciplinary project [26, 27]. Two horizons were collected from core 205-S6 (Comacchio, 44°68’N, 12°15’E), code “CO1” and “CO2”. The third sub-fossil horizon (code “CE”) was collected from core 240-S8 (Cervia 44°16’N, 12°20’E) (Fig 1). All investigated horizons came from shoreface depositional environments characterized by sandy substrates and estimated water depth between ~5 and 10 m. Paleoenvironmental, paleobathymetric and paleogeographic reconstructions of the Po-Adriatic system during the Holocene are detailed in previous studies [19, 27–29] (for details see S1 Text “Geological setting” in S1 File).

Modern samples of *C*. *gallina* were collected in the Northern Adriatic Sea off the coast of Goro (MGO; 44°75’N, 12°43’E) and Cervia (MCE; 44°30’N, 12°40’E) (Fig 1). Samplings were performed by means of Van Veen Grab and scuba diving on the sandy bottom at 5.2 m and 5.3 m water depth. The sampling areas are about fifty kilometers away from each other and correspond roughly to the extraction areas of cores used in this study. Sampling operations were restricted to the top-most 10 cm of the taphonomically active zone (TAZ) of the sea bottom. This sampling allowed to collect a time-averaged record of shells estimated in tens of years, following the deposition rates reported in Trincardi *et al*. [30]. This allowed a better comparison with cored sub-fossil horizons, in which the sampled shells came from a time span of tens of years. No living organism was collected for this study. The specimens are housed at the Department of Biological, Geological and Environmental Sciences (Bologna, Italy), repository number MGGC 26131 and MGGC 26138(for samples from cores CE and CO, respectively) and MGGC 26350 for samples from modern thanatocoenosis. Shell data used in this study have been archived as a PLoS One online-access appendix (S1 Appendix).

Only valves of 5–30 mm length (maximum distance on the anterior-posterior axis) were considered for the analyses. The lower limit was defined by the technical difficulties in obtaining reliable measurements in very small specimens. The upper limit was due to the difficulty to collect whole shells over 30 mm in the sub-fossil horizons with 90 mm cores diameter and in finding valves over 30 mm in thanatocoenoses located in *C*. *gallina* harvesting areas.

Prior to any measurements, each valve was cleaned with a toothbrush and soaked in distilled water for two hours to remove any external residue on the shells surfaces. In addition, valves from modern Adriatic settings were immersed in a solution of distilled water and hydrogen peroxide (5 vol.%) for 24 h to eliminate any traces of organic material on the surface (e.g., epibionts). Then, the valves were dried in an oven at 37°C for one night to remove any moisture that may influence subsequent measurements.

### Radiocarbon measurements

A dating was performed exploiting the high-resolution stratigraphic framework developed for the Holocene succession of the Po coastal plain, which allowed to subdivide this ca. 30 m thick sedimentary package in millennial-scale stratigraphic units (parasequences in [13]). Successively, radiocarbon dating was performed on five randomly selected valves to constrain the time span of the examined sample. Radiocarbon data were calibrated with Oxcal 4.2 [32], using the Intcal13 calibration curve [33], Delta R (ΔR) = 139.0 ± 28.0 and obtained from CHRONO Marine Reservoir Database, Map No 235 (North Adriatic, Rimini, Italy).

### Environmental parameters

Sea surface temperature (SST) for the Adriatic Sea in proximity of targeted shoreface settings were obtained from the global ocean OSTIA sea surface temperature and sea ice analysis databank [34]. Mean annual SST was calculated from daily values measured from January 2010 to December 2019 (number of daily values = 3651 for each site).

As for sub-fossil *C*. *gallina* horizons, SST estimates were based on Alkenones unsaturation index, a widely applied proxy for past SST. Alkenones are long-chain methyl ketones synthesized by some single-celled algae found in marine sediments and whose carbon bond saturation index varies according to annual mean values at the SST [35]. Jalali *et al*. [36] produced a high-resolution SST record of the past 10,000 years based on alkenone paleothermometry for the central-northern Mediterranean Sea (Gulf of Lion). This site is at the same latitude of the North Adriatic Sea and shows a comparable physiographic setting. Estimated paleo-SST for the Gulf of Lion can be considered a reliable proxy for the study area too. Mean SST estimated for the Gulf of Lion (off Leucate) from January 2010 to December 2019 is 16.7 ± 0.1°C.

### Shell parameters

Shell length (maximum distance on the anterior-posterior axis) and height (maximum distance on the dorsal-ventral axis) were measured using ImageJ software after data capture of each shell shape with a scanner (Acer Acerscan Prisa 620 ST 600 dpi). The shell width (maximum distance on the lateral axis of the valve) was measured with a caliper (± 0.05 mm).

Skeletal parameters were measured by buoyant weight (BW) analysis, using a density determination kit Ohaus Explorer Pro balance (± 0.1 mg; Ohaus Corp., Pine Brook, NJ, USA, see Gizzi *et al*. [24] for details).

The BW measurement was repeated three times and the average was considered for statistical analysis. The BW technique allowed to estimate the variable of interest:

- micro-density or matrix density (mass per unit volume of the material which composes the shell, excluding the volume of pores; g·cm^−3^);
- porosity: the volume of pores connected to the external surface (%);
- bulk density: the density of the valve (including the volume of pores).

Correlations analyses between SST and skeletal parameters were performed to investigate any significant pattern developed over geological time as a function of temperature.

Differences in skeletal properties of *C*. *gallina* shells were also investigated in relation to animal sexual maturity (reached in modern specimens after 1 year of life [23] and length >18mm) in order to consider eventual differences in the biomineralization process during different stages of the bivalve’s life cycle.

### Shell phase composition and microstructure

Nano-scale and micro-scale analyses of skeletal features were used to determine the mineral phase and an eventual recrystallization or alteration of the samples.

Prior to the analyses, samples were soaked in an ethanol solution (10 vol.%) and immersed in a bath sonicator (Falc Instruments S.r.l., UTA 18) for one minute. Subsequently, the valves were treated with a sodium hypochlorite solution (5 wt.%) for one hour, rinsed with distilled water and dried in a desiccator. About one-half of each shell was finely grounded in a mortar to obtain a homogenous powder.

X-ray powder diffraction (XRD) analyses were performed on six specimens for each horizon, by preparing a thin compact layer of the sample in a silica background signal free holder. Diffractograms for each sample were collected using an X’celerator detector fitted on a PANalytical X’Pert Pro diffractometer, using a Cu-Kα radiation generated at 40 kV and 40 mA. The data were collected within the 2θ range from 20° to 60° with a step size (Δ2θ) of 0.016° and a counting time of 60 s. Fixed anti-scatter and divergence slits of 1/2° were used with 10 mm beam mask. All measurements were carried out in a continuous mode. The XRD patterns were analyzed using the X’Pert HighScore Plus software (PANalytical).

High-resolution synchrotron X-ray powder diffraction (HR-XRPD) measurements were performed on three valves of the oldest horizon (CO1) and three of today’s thanatocoenosis (MCE). The analysis was carried out on ID22 beamline at the European Synchrotron Radiation Facility (ESRF) in Grenoble, France, using a monochromatic radiation of 0.49599 Å. Each sample was transferred to a 0.9 mm glass capillary and measured three times at a fast rate (10 deg·min^-1^) at three different locations, while being rotated. This setup makes it possible to avoid beam damage and texture influences. Measurements were performed at room temperature and after *ex-situ* heating at 300°C for 2 h in order to examine possible influence of the intracrystalline organics on the shell’s unit cell. The unit cell parameters were extracted using Rietveld refinement method applied to a full diffraction pattern profile. Coherence length (nm) along specific crystallographic directions was derived by applying the line profile analysis to a specific diffraction peak. This was performed by fitting the diffraction peak profile to a Voigt function and deconvolution of the diffraction peak broadening into the Lorenzian and Gaussian widths.

Fourier-transform infrared spectroscopy (FTIR) analyses were performed on twelve valves for each site using a Nicolet IS10 Spectrometer (Thermo Electron Corporation) working in the 4,000–400 cm^-1^ range of wave numbers at a resolution of 2 cm^-1^. The samples were analyzed as KBr pellets using a sample concentration of about 1 wt.%.

Thermogravimetric analysis (TGA) was used to estimate the organic matrix (OM) and the structurally associated intra-skeletal water content of each shell. The measurements were performed using a SDT Q900 instrument (TA Instruments). Five different valves were analyzed for each horizon, by measuring 10–15 mg of sample in a ceramic crucible. The analysis was carried out under nitrogen flow with a pre-equilibration at 30°C, followed by a heating ramp from 30°C to 850°C using a 10°C·min^-1^ heating rate.

Inductively coupled plasma optical emission spectroscopy (ICP-OES) measurements to evaluate the metal content of shells were performed on valves treated with sodium hypochlorite (5 wt.%) for 24 h, then rinsed with distilled water and dried in a desiccator. About 1 g of shell was dissolved in 3 mL of HCl and HNO_3_ in a 1: 3 volume ratio, adjusting the volume with milliQ water until 5 mL. Solvents and reagents with trace analysis grade of purity were used. Three samples were measured for each level. Each sample was measured three times, 12 s each with 50 s of prerunning, using a Spectro Arcos-Ametek, ICP-OES with axial torch and high salinity kit.

### Statistical analyses

Levene’s test was used for testing homogeneity of variance while Kolmogorov-Smirnov’s test was used for testing normality for environmental and shell parameters. Since assumptions for parametric statistics were not fulfilled, the non-parametric Kruskal-Wallis equality-of-populations rank test was used. Spearman’s rank correlation coefficient was used to evaluate trend between shell parameters and sea surface temperature. In each horizon, rank-correlations were computed on all valves and also on two subgroups consisting of immature specimens (valve length <18 mm) and mature ones (>18 mm [23]). All statistical analyses were computed using RStudio software [37].

## Results

### Dating and environmental parameters

Radiocarbon measurements ascribed two of the sub-fossil horizons to the Middle Holocene (CO1 and CO2) and one to the Late Holocene (CE) as reported in Table 1.

**Table 1:** Calibrated radiocarbon age, Sea Surface Temperature (SST), shell biometric and skeletal parameters.

Horizon	n	^14^C Age (ky BP)	SST (°C)	Length (mm)	Height (mm)	Width (mm)	Mass (g)	Micro-density (g/cm3)	Apparent Porosity (%)	Bulk Density (g/cm3)
CO1	49	7.6 ± 0.1	18.6 ± 0.4	17.6 ± 0.9	14.2 ± 0.7	4.7 ± 0.2	0.66 ± 0.07	2.81 ± 0.01	10.17 ± 0.55	2.52 ± 0.02
CO2	59	5.9 ± 0.1	18.2 ± 0.3	17.2 ± 0.8	14.8 ± 0.7	4.3 ± 0.2	0.74 ± 0.09	2.79 ± 0.01	8.58 ±0.42	2.55 ± 0.01
CE	52	2.6 ± 0.2	17.5 ± 0.5	15.7 ± 0.7	13.6 ± 0.6	3.9 ± 0.2	0.47 ± 0.06	2.80 ± 0.01	10.28 ± 0.72	2.52 ± 0.02
MCE	73	modern	17.3 ± 0.1	16.7 ± 0.8	13.6 ± 0.6	4.0 ± 0.2	0.56 ± 0.06	2.78 ± 0.01	9.87 ± 0.34	2.51 ± 0.01
MGO	68	modern	17.2 ± 0.1	17.3 ± 0.8	14.7 ± 0.7	4.3 ± 0.2	0.67 ± 0.08	2.78 ± 0.01	11.01 ± 0.59	2.47 ± 0.02
KW			***					***	*	***

Values for each horizon in chronological order. Radiocarbon measurements are reported in years `before present’ (BP). Holocene SST are extrapolated from Jalali *et al*. [36]. For each parameter mean value and standard error are reported. n  =  number of collected specimens. K-W  =  Kruskal-Wallis equality-of-populations rank test;

* p < 0.05,

*** p < 0.001.

According to the data reported for the Gulf of Lion, estimated and measured SST appeared to cool down gradually moving from the oldest horizon (CO1, 18.6°C) to nowadays setting (MCE, 17.3 and MGO, 17.2°C) (Kruskal-Wallis test, df = 4, p < 0.001; Table 1). The reconstructed SST trend for the Holocene showed a difference of ∼1.5°C between middle Holocene and present day, a difference that is comparable with the current SST variation along the latitudinal gradient in the Adriatic Sea (Gizzi *et al*. [24]).

### Shell parameters

All the measured shell parameters (*i*.*e*., length, height, width and mass; Table 1) were homogeneous among horizons (Kruskal-Wallis test, p > 0.05, Table 1). In all investigated *C*. *gallina* assemblages, length correlated positively with height, width, and mass (S1 Fig in S1 File). Shell length correlated with skeletal parameters (*i*.*e*., bulk-, micro-density and apparent porosity) except for apparent porosity and length of levels CO2 and MCE (S1 Fig in S1 File). Skeletal parameters resulted significantly different among stratigraphic horizons both in the whole dataset and in the subgroups (*i*.*e*., mature and immature shells) (Tables 1 and 2).

**Table 2:** Skeletal parameters for immature and mature shells.

		Horizon	^14^C Age	SST	Micro-density (g/cm^3^)	Apparent Porosity (%)	Bulk Density (g/cm^3^)
			(ky BP)	(°C)			
**Immature shell**	**(≤18 mm)**	CO1	7.6 ± 0.1	18.6 ± 0.4	2.78 ± 0.014	11.81 ± 0.8	2.45 ± 0.03
		CO2	5.9 ± 0.1	18.2 ± 0.3	2.77 ± 0.006	8.89 ± 0.7	2.52 ± 0.02
		CE	2.6 ± 0.2	17.5 ± 0.5	2.79 ± 0.007	12.04 ± 1.0	2.46 ± 0.03
		MCE	modern	17.3 ± 0.1	2.77 ± 0.004	10.67 ± 0.5	2.47 ± 0.01
		MGO	modern	17.2 ± 0.1	2.76 ± 0.004	13.54 ± 0.9	2.39 ± 0.03
		KW		***	***	***	***
**Mature shell**	**(>18 mm)**	CO1	7.6 ± 0.1	18.6 ± 0.4	2.84 ± 0.004	8.60 ± 0.6	2.59 ± 0.02
		CO2	5.9 ± 0.1	18.2 ± 0.3	2.82 ± 0.002	8.24 ± 0.4	2.59 ± 0.01
		CE	2.6 ± 0.2	17.5 ± 0.5	2.82 ± 0.002	6.94 ± 0.4	2.63 ± 0.01
		MCE	modern	17.3 ± 0.1	2.80 ± 0.003	8.86 ± 0.3	2.55 ± 0.01
		MGO	modern	17.2 ± 0.1	2.79 ± 0.002	8.27 ± 0.3	2.56 ± 0.01
		KW		***	***	***	***

Values for each horizon in chronological order. For each parameter mean value and standard error are reported. Holocene SST are extrapolated from Jalali et al. [36]. K-W  =  Kruskal-Wallis equality-of-populations rank test;

*** p < 0.001.

**Fig 2:**
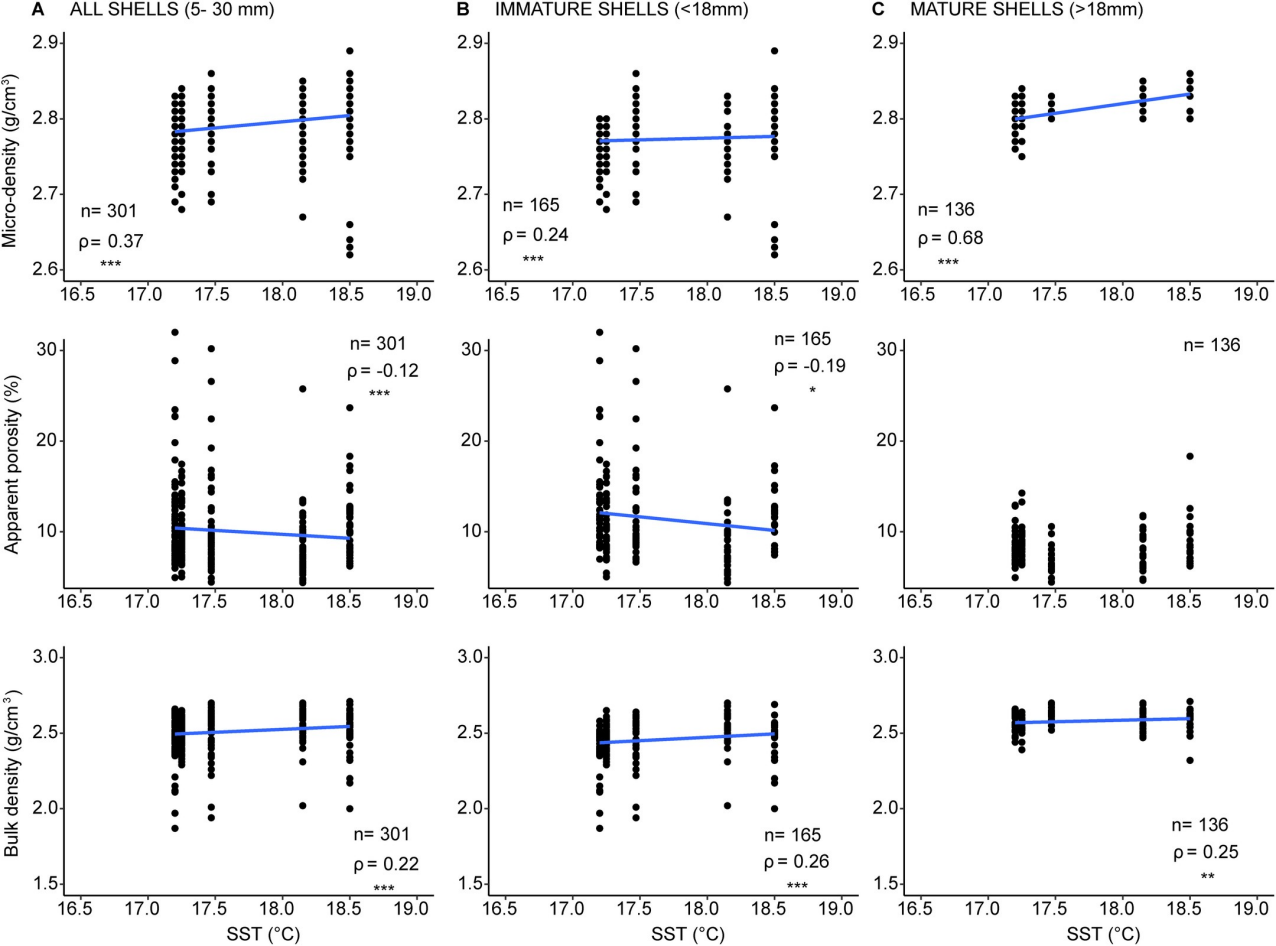
Relationship between shell skeletal parameters and Sea Surface Temperature (SST). (A): whole dataset, (B): immature shells and (C): mature shells. n = total specimens analyzed; ρ =  Spearman’s determination coefficient; * p < 0.05; ** p < 0.01; *** p < 0.001.

In both cases, micro- and bulk density were positively correlated with SST, while apparent porosity correlated negatively with SST (Fig 2). The only exception was represented by the subgroup of mature shells, which showed no significant correlation between apparent porosity and SST (Fig 2C).

### Shell phase composition and microstructure

**Fig 3:**
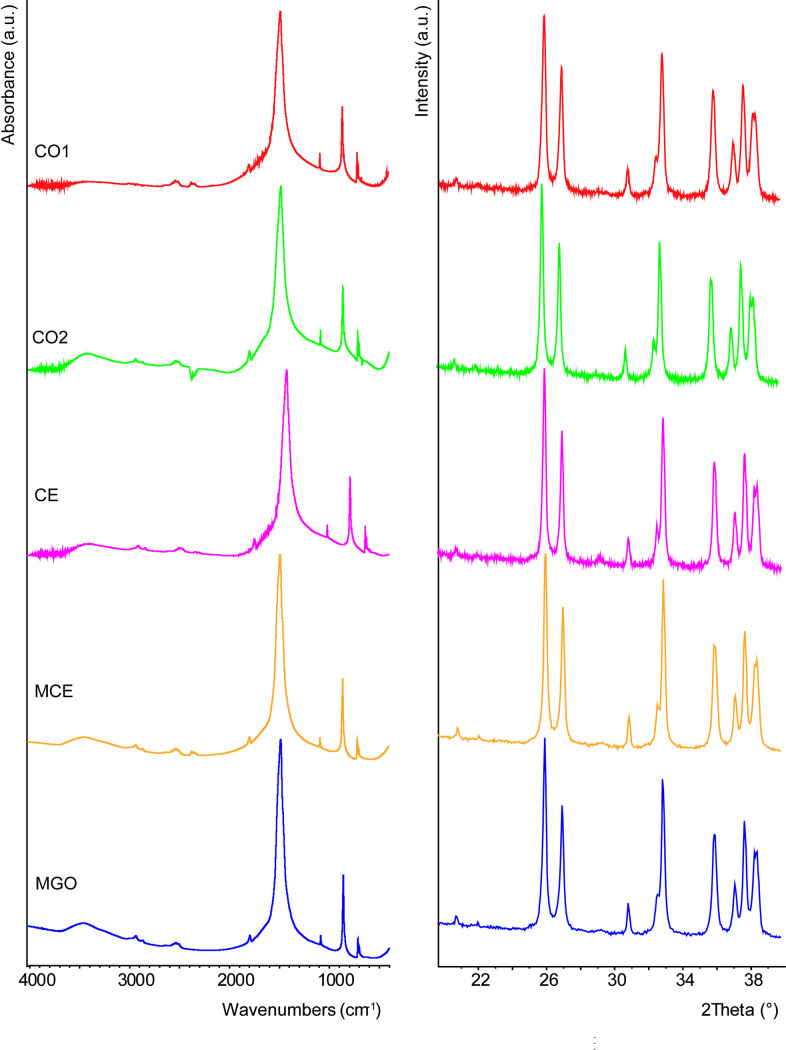
Fourier-transform infrared (FTIR) spectra (on the left chart) and X-ray powder diffraction (XRD) patterns (on the right chart) from grinded valves of *C*. *gallina*. A representative diffraction pattern and FTIR spectrum is shown for each level from the older samples (top) to more recent ones (bottom).

**Fig 4:**
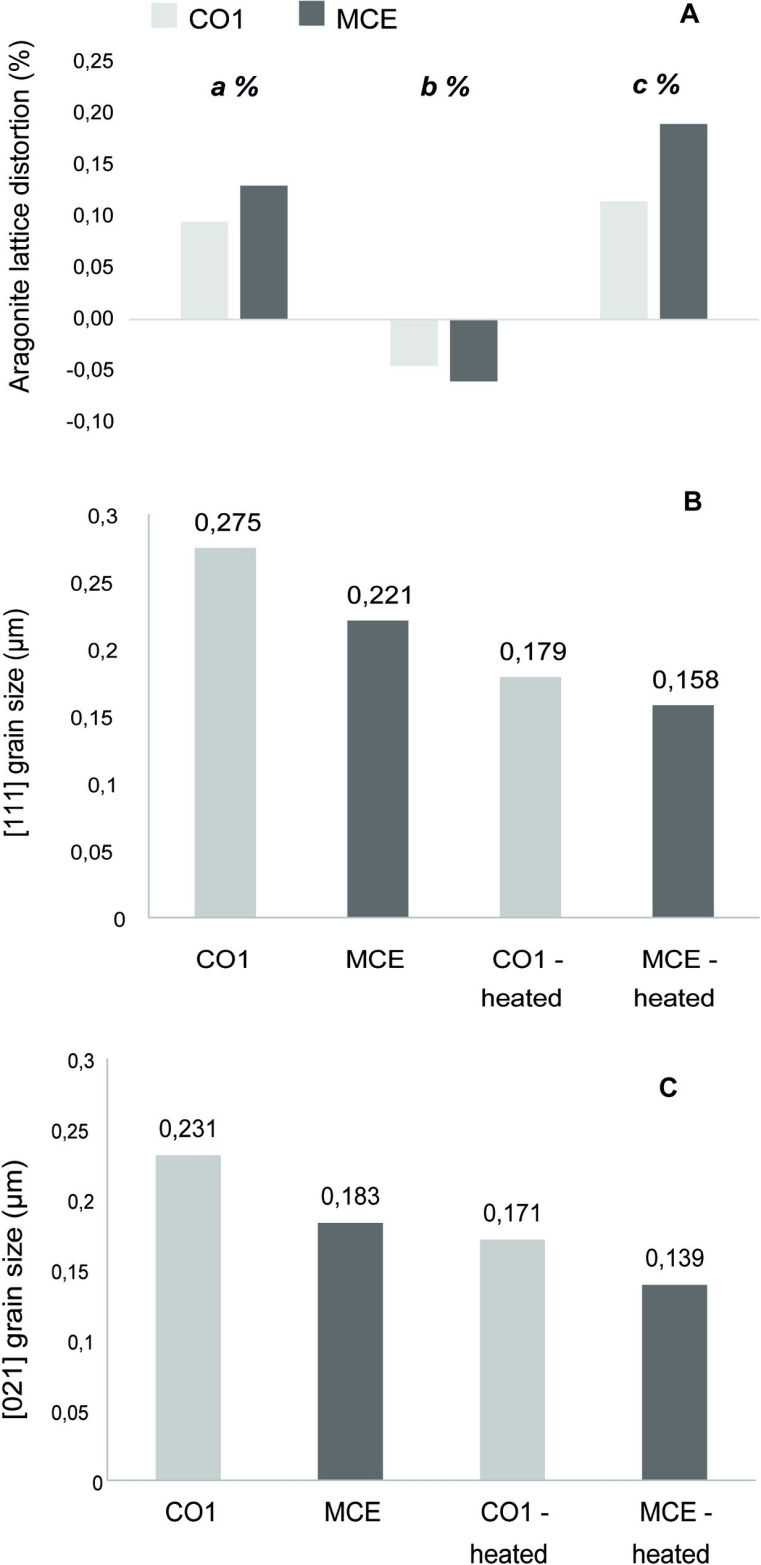
HR-XRPD data. (A) Distortions along a, b, and c axis of aragonite due to the presence of the intraskeletal organic matrix. Crystallite sizes of aragonite along the (B) (111) and (C) (021) directions, respectively, of pristine and thermal-treated samples.

The conventional XRD and FTIR analyses (Fig 3) of the shells from all levels showed only aragonite signals, no other mineral phase was detected. However, the HR-XRPD data (S2 Fig in S1 File) allowed to precisely deduce the unit cell parameters, microstrain fluctuations and crystallite size. The heat treatment removed possible effects of the OM on the unit cell of the shells. The obtained data revealed that the intracrystalline OM induced an elongation of both the *a*- and *c*-axes and a contraction of the *b*-axis (Fig 4A). Values of the calculated lattice distortions vary from 0.15% to 0.20%, with the highest strain observed in the case of the modern sample (MCE). The line profile analysis allowed to derive the crystallite sizes along the <111> and <021> aragonite directions for the MCE sample, 0.221 and 0.183 μm, and CO1 samples, 0.275 and 0.231 μm, respectively. After the thermal treatment, the crystallite sizes were 0.158 and 0.139 μm for the MCE, and 0.179 and 0.171 μm for the CO1, respectively (Fig 4B and 4C).

The shell’s skeletal weight loss measured by TGA before aragonite decomposition, in the temperature range between ~ 150 and 450°C, differed among horizons (p< 0.05), but no correlation with SST was found (S1 Table in S1 File). The weight percentage of the OM and the associated intra-skeletal water was slightly lower in the case of the sub-fossil horizons, where the values varied from 1.37 to 1.72%, as compared to that of the both modern horizons with 1.79 and 1.83%. (S1 Table in S1 File).

The metal content analysis (ICP-OES) showed no differences between the sub-fossil and modern horizons in the content of magnesium and strontium, two elements that may vary in response to a diagenetic alteration occurred over time [38] (S1 Table in S1 File).

## Discussion

This study investigated the phenotypic variation of *C*. *gallina* in relation to SST trend in the Po Plain-Adriatic Sea system during the last ~8,000 years. By comparing the Holocene sub-fossil record to modern day thanatocoenoses, has been possible to get insight on skeletal dynamics on a millennial temporal scale. This allowed overcoming the time limits imposed by laboratory studies and assessing how rising SST and environmental-driven changes could affect this economically relevant bivalve in the future. The high paleontological and stratigraphic resolution of the investigated succession offered an ideal venue to explore this scenario.

On a geologic time scale, the taphonomic status of a skeletal remain could be an indicator of its relative age [39]. This concept goes under the name of taphonomic clock [40]. Although intriguing, the taphonomic clock shows a variable reliability, as it is not only function of time since-death, but mainly depends on the time spent by the skeletal remain in the taphonomic active zone (TAZ) of the sediment layer, where it is exposed to physical and biological degradation processes [41]. In Holocene sub-fossils, most distinctive external features related to taphonomic alterations as a function of time would be expressed as lack of color, chalky surface or loss of glossiness. Other than aesthetic damages, deterioration of fossil remains also affects the preservation of the mineral phase that constitutes the shell. Indeed, in fossil records of biogenic calcium carbonate biominerals a partial or complete recrystallization might occur over time. This process might lead to recrystallization of aragonite into a more stable polymorph, such as calcite, or different minerals, such as calcium phosphate [42].

Overall, a sustained variation in the mineral composition could deeply alter the original organization of the biomineral phase and in the end be responsible for spurious trends. In this study, if external and internal taphonomic degradations had occurred, older shells would have a chalky surface and would show a reduction in micro-density of shells, since calcite has a lower density than aragonite (2.71 mg·mm^-3^ vs 2.94 mg·mm^-3^ [43]). On the contrary, our data report higher shells micro-density values in the most ancient horizons than in modern ones. Moreover, the application of both HR-XRPD and FTIR detected no other mineral phase than aragonite (Fig 3), the original mineralogy of *C*. *gallina* shell. Thus, we can assume no recrystallization process occurred in the geological time period examined.

The analyses of the HR-XRPD data allowed also to quantify the strain with the crystals due to the presence of the intra-crystalline OM. The obtained data on the lattice distortions and micro-structural parameters (microstrain fluctuations and crystallite size) were in line with those reported in the literature for the biogenic aragonite from other organisms [44, 45]. The lattice strain was lower in the sub-fossil samples (CO1) compared to the modern ones (MCE; Fig 4), indicating a partial degradation of the organic matrix in the sub-fossil samples. The values of the crystallite size after the thermal treatment, which is known to lead to the removal of the OM [44, 45], were lower than those of the non-treated samples and were quite similar for the both MCE and CO1 samples. The latter further confirms the presence of OM and supports its role in determining the lattice strain (Fig 4). Thus, we can safely state that in the sample CO1 the OM were still present, excluding a relevant re-crystallization of aragonite crystallite that should imply a loss of the strain, even if partially degraded. Moreover, we could speculate that degradation processes occurred mainly in the inter-crystallite fraction of the organic matrix rather than in the intra-crystallite one.

The fact that no recrystallization process occurred in the sub-fossil shells was also confirmed by the values of the measured metal content that were constant among all samples, excluding important environmental fluid diffusion into the biomineral. This result was in agreement with previous studies reporting that in marine shallow settings certain parameters, such as high sedimentation rates, could rapidly sequester skeletal remains from the TAZ, increasing their preservation [20]. In conclusion, for the purpose of our study, these evidences allowed to rule out the possible influence of the taphonomic alteration of the mineral phase on the observed trends of the skeletal parameters.

**Fig 5:**
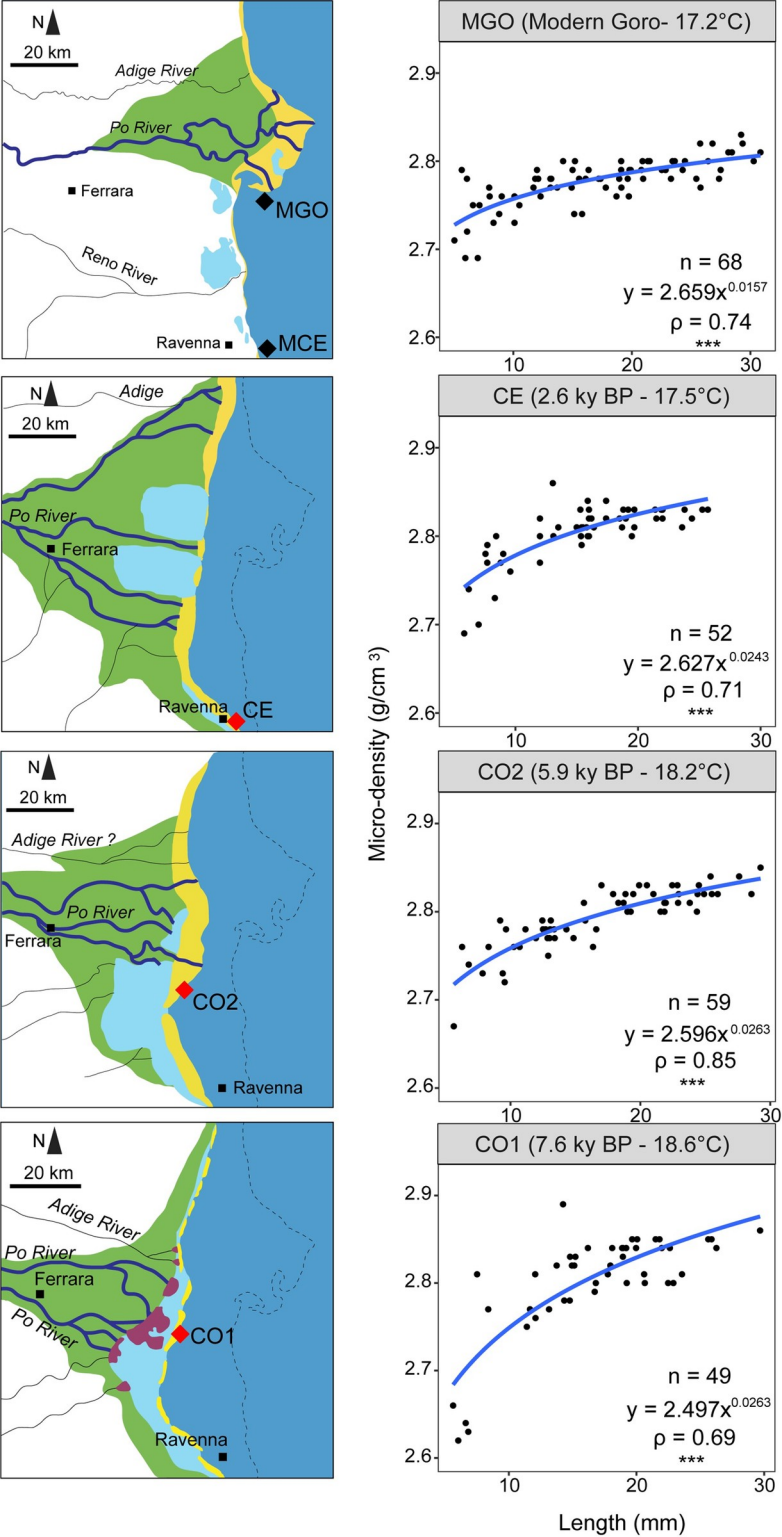
Po coastal plain changes through time and variation in micro-density recorded within the five horizons investigated. Of the two modern settings, only Goro (MGO) data are reported since they show comparable patterns and SSTs (all analyses are shown in S1 Fig in S1 File). The dotted line represents the modern coastline. n = number of valves; ρ = Spearman’s determination coefficient; * p < 0.05; ** p < 0.01; *** p < 0.001. Geomorphologic map after Amorosi *et al*. [13, 14], slightly modified and for illustrative purposes only.

*C*. *gallina* skeletal parameters differed between mature and immature clams (Fig 2 and S1 Fig in S1 File) in their biomineralization patterns. Higher apparent porosity was observed both in sub-fossil and modern horizons for shells of small size, decreasing from more than 20 to less than 15% approaching the length at sexual maturity (about 18 mm [23]). High porosity influenced bulk density, which was conversely lower in small size shells. Micro-density followed the same pattern as bulk-density. This trend agreed with previous study carried out in living populations of *C*. *gallina* from the Adriatic Sea [25]. Hence, suggesting that Middle Holocene specimens of *C*. *gallina* in different climate-environmental contexts (Fig 5 and S1 Fig in S1 File) exerted a similar physiological control on biomineralization of calcium carbonate during their lifespan. In agreement with Mancuso *et al*. [25], mature specimens of *C*. *gallina* seemed to change their biomineralization behavior, showing small variations in apparent porosity and bulk density compared to immature ones. This suggested that *C*. *gallina* promoted an accelerated shell accretion, in order to quickly reach the size required for sexual maturity, at the expense of possessing a less dense, more porous and mechanically weaker shell. Apparent porosity showed no correlation with SST in mature shells and a significant negative correlation in immature shells (Fig 2). Bulk and micro-density increased with increasing SST, for both mature and immature shells. The significant correlation in shell density with SST can be attributed to different mineralization rates driven by temperature and related to aragonite saturation state. Warmer water masses reduce the thermodynamic work required to organisms to deposit calcium carbonate [46, 47], making the calcification less expensive in terms of metabolic cost [48]. This enables an increase in calcification rates [49]. Comparable patterns have been detected also in brachiopods, where some species living in cold water showed a reduced calcium carbonate deposition and an increased organic matrix content compared to higher temperate settings, characterized by larger crystals and reduced organic matrix (hence denser shells [50]).

Previous studies on *C*. *gallina* shells were conducted along a latitudinal gradient in the Adriatic Sea, including the area considered in this study [24, 25]. Mature shells of *C*. *gallina* of commercial size over 25 mm long were thinner, more porous and less resistant to fractures in warmer and more irradiated populations [24, 25]. On the other hand immature shells, less than 18 mm long, showed the opposite trend to mature ones, resulting in more porous and less dense shells with lower SST [25] (S3–S5 Figs in S1 File). According to these results, local environmental parameters seemed to have a different influence on the biomineralization rate of mature shells compared to immature ones, likely due to different growth and metabolic rates [25, 51]. This might suggest that while immature clams have an energy surplus to better withstand environmental stress, mature clams are more dependent on their reserves [52].

Studies on mollusks and other macroinvertebrates highlight that calcification increases with aragonite saturation state [48, 49]. Trends depicted here conform to those patterns of biomineralization in warmer settings. However, no aragonite saturation data were available along the considered temporal gradient to investigate relationships between shell calcification and seawater chemistry in the studied area.

Although all samples were collected in the same area, a strong geomorphological change took place during the Holocene in response to the glacio-eustatic sea-level variations [14]. The highest values for micro-density and bulk density of *C*. *gallina* shells were recorded during the Holocene climate optimum (HCO, 9–5 ky BP), when SSTs in the study area were higher than today. The North Western Adriatic coastal area was characterized by estuary systems, bounded seaward by a series of sandbars that isolated coastal lagoons and limited riverine plumes into the Adriatic (Fig 5) [14]. Mancuso *et al*. [25] reported that *C*. *gallina* populations could be negatively impacted by riverine influence (*i*.*e*., reduction of net calcification and linear extension rates). The positive correlation between temperature and shell density of *C*. *gallina* specimens found in the current study could be facilitated, other than higher aragonite saturation state due to past warmer conditions, by a more stable shoreface depositional setting due to reduced influence of riverine plumes. Indeed, in estuarine system mixing between freshwater and marine water occurs in the back barrier settings and not in the shallow marine zone where *C*. *gallina* thrives. By contrast, during the last part of the HCO, the weight of eustasy on the coastal dynamics of the study area largely vanished, and the study area transitioned (between 7.0 to 2.0 ky BP) to a wave-dominated and, after 2.0 ky BP, to a river-dominated deltaic system [14]. The last geomorphologic configurations led to progressively increasing influence of riverine processes on the control of coastal dynamics and the storage-release of sediments [53]. The enhanced freshwater discharge in the nearshore area, especially during the last 2.0 ky BP, resulted in a strong progradation and the upbuilding of the modern Po Delta [14, 54] in a climatic context characterized by an overall decreasing trend in SST. The upbuilding of the modern Po Delta likely helped the installation of a low temperature and salinity wedge in the coastal area around it [55] and southward, due to action of anti-clock wise long-shore currents. Indeed, during flood events the modern Po river plume can influence the sea facing area in a radius of ~60 km [56]. Freshwater plumes can reduce the SST between 2°C to 6°C, with a sensible effect down to 10 m of depth [57]. The drop in SST could have reduced the aragonite saturation state in the seawater, increasing the metabolic cost for calcification of *C*. *gallina*. Moreover, although *C*. *gallina* is euryhaline, the installation of suboptimal salinity level due to the riverine inflows could lead to a reduced feeding activity and slower net calcification rates, as documented also for other macrobenthic species [24, 25, 58]. Additionally, the recorded decline of shell density could also be associated to the increasing water turbidity and oligotrophic conditions as the Po Delta advanced into the Adriatic Sea. During the early HCO, the estuary-lagoon acted as a material sink, accommodating most of the sediments and nutrients debouched by the Po River [14, 59]. This settings likely reduced the water turbidity, with positive repercussions on feeding activity [60], providing *C*. *gallina* with spare metabolic energy to sustain higher net calcification and linear extension rates [61]. By contrast, during the onset of a wave dominated and then a fluvial dominated deltaic system the sediment and nutrients runoff directly into the shallow Adriatic Sea progressively increased [14]. The resuspension of fine bottom sediments, could have increased the turbidity with serious consequences on the feeding activities of bivalves by reducing the rate of water pumped, increasing the period of valve closure [25, 62] and damaging bivalves gills [60], overall cutting the energy available for the skeletal construction.

The discrepancy between this and previous works in the shell density for mature clams (positive correlation with SST found in this study, VS negative correlation with SST found in Gizzi *et al*. [24] and Mancuso *et al*. [25], S4 Fig in S1 File) suggested that this parameter is not only dependent on physical environmental factors (SST, salinity, aragonite saturation, sediments and nutrient supply), but is affected by a complex interplay between physical, biological and physiological factors, making clams response less predictable to changing environmental parameters. When making this comparison we assume that past and modern clams belong to the same species, without large spatial and temporal variability. As reported by Papetti *et al*. [63], modern populations of *C*. *gallina* in northern-central Adriatic Sea are homogeneous at large geographic scale, displaying low genetic differentiation at local and temporal scales. Variability of local circulation, reproductive success, and high larval mortality rates are recognized as the main factors determining the negligible genetic differences observed today [63]. Nevertheless, although the present-day situation suggests a rather homogenous genetic structure, we cannot exclude that in the past this variability was larger than today and that the trends observed in this study may reflect environmentally-driven migration of different *C*. *gallina* morphotypes.

Moreover, since 2.0 ky BP, the anthropogenic influence on the Po Delta evolution constantly grew becoming dominant around the 17^th^ century when river diversion and channels stabilization led to the growth of the modern Delta. These human interventions dictated an increase in sediments runoff, eutrophication events and anoxic events, overall contributing in increasing the instability and stress of the nearshore environments, whose effects on *C*. *gallina* skeletal construction cannot be excluded. On a millennial time scale, temperature can be considered as a complex gradient that not only affects skeletal biomineralization directly by exerting a physiological response but also indirectly, by influencing the geomorphologic configuration and environmental parameters of *C*. *gallina* biotope.

## Conclusion

*Chamelea gallina* shells appeared to be sensitive to changes in seawater temperature. At the macroscale level, specimens from past sub-fossil horizons, living in warmer water, presented a denser, less porous shells than modern specimens. The significant correlation between temperature and skeletal density remained consistent even when dividing the total dataset into two minor subgroups and analysing immature and sexually mature individuals, separately.

At the microscale level, the shells were all composed of pure aragonite, presenting a perfectly preserved mineral phase with no relevant diagenetic alteration and only a slight degradation of the inter-crystalline organic phase. Hence, the observed difference in micro-density is not ascribable to any of the parameters here measured. Other factors not investigated in this study, such as occluded porosity and intra-crystalline water content, may be at the origin of the observed differences.

This study along a temporal gradient represented a complementary approach to previous studies conducted along a latitudinal gradient in the Adriatic Sea and together improved our understanding of the response of this economically relevant species to a changing environment in face to seawater warming.

## Supporting information

S1 File (DOCX)

S1 Appendix *Chamelea gallina* dataset. (XLSX)
