# Supplementary material for: Climate variation during the Holocene influenced the skeletal properties of Chamelea gallina shells in the North Adriatic Sea (Italy)
Source: PLoS One. 2021 Mar 4;16(3):e0247590. doi: 10.1371/journal.pone.0247590 (PMC7932108; doi:10.1371/journal.pone.0247590)
Supplement: S1 File — (DOCX) [file pone.0247590.s001.docx]

**S1 File.**

**Geological setting**

The Po coastal plain treasures records of past environmental shifts that reflect the complex interplay between climate driven sea-level changes and coastal dynamics [1,2]. Stratigraphic paleobiology investigations over the past years led to a high-resolution framework of the study area in term of environmental and biological dynamics, here briefly sketched. The latest Quaternary (<30 ky BP) succession of the Po coastal plain is a few tens of meters thick (Fig 1). This succession displays a wedge-shaped package of coastal and marine units recording the recent interplay between local sediment supply and accommodation space.

The fluvial-channel and associated floodplain deposits of the lower part of the succession are interpreted to record progradation and aggradation during the last glacial maximum and the onset of sea-level rise (<14.5 ky BP). At that time the Po Delta was located near the Mid-Adriatic Deep and the Northern Adriatic was entirely subaerially exposed [3]. In the study area, these glacial and early transgressive alluvial and marsh deposits are overlaid by a few meters of shallow marine fossil rich sandstones forming elongated shoreline-parallel barriers and delimiting inland a back-barrier brackish zone and bay-head deltas in correspondence of the estuaries fluvial mouths [4]; Fig 1). These elongated shoreface-related bodies recorded short-lived intervals of rapid sedimentation [5] linked to sea-level stillstand phases in the overall post-glacial transgression of the Adriatic shelf. After the transgression reached its peak and in response to a deceleration of the sea-level rise (~7.0 until 2.0 ka BP) an early phase of slow progradation of bay-head deltas (ca. 5 m/year), initially filled great part of the back barrier area and then, by delta-lobe switching processes and sediment redistribution, built mainland attached and continuous beaches (Fig 1). Hence, during the middle Holocene the physiography of the study area transitioned from a barrier-lagoon-estuary system to wave dominated coastal and deltaic systems [6]. These environmental dynamics are recorded in the sedimentary succession by aggradational to slightly progradational stacking of beach and barrier sandy units characterized by shallowing upward trends [2,7–9], passing inland to floodplain and wetland deposits. During this phase, the distal part of Po-Adriatic deltaic system continued to experience a phase of sediment starvation due to the trapping of coarse-grained sediments in the nearshore area and around the river outlets [5,6]. From 2.0 ka BP onward, the deltaic and coastal systems of Emilia-Romagna experienced a period of increased rivers discharge that promoted a strong progradation phase and the transition from wave-dominated to river-dominated deltaic systems shifting spatially due to multiple episodes of river avulsion. The last 800 years record the up building of the present-day morphological configuration. In 1152 AD the last natural Po river major avulsion occurred. The Po delta shifted northward respect to its previous location and during the last 400 years built its present-day morphological configuration [10].

**S1 Text. Geological Setting**


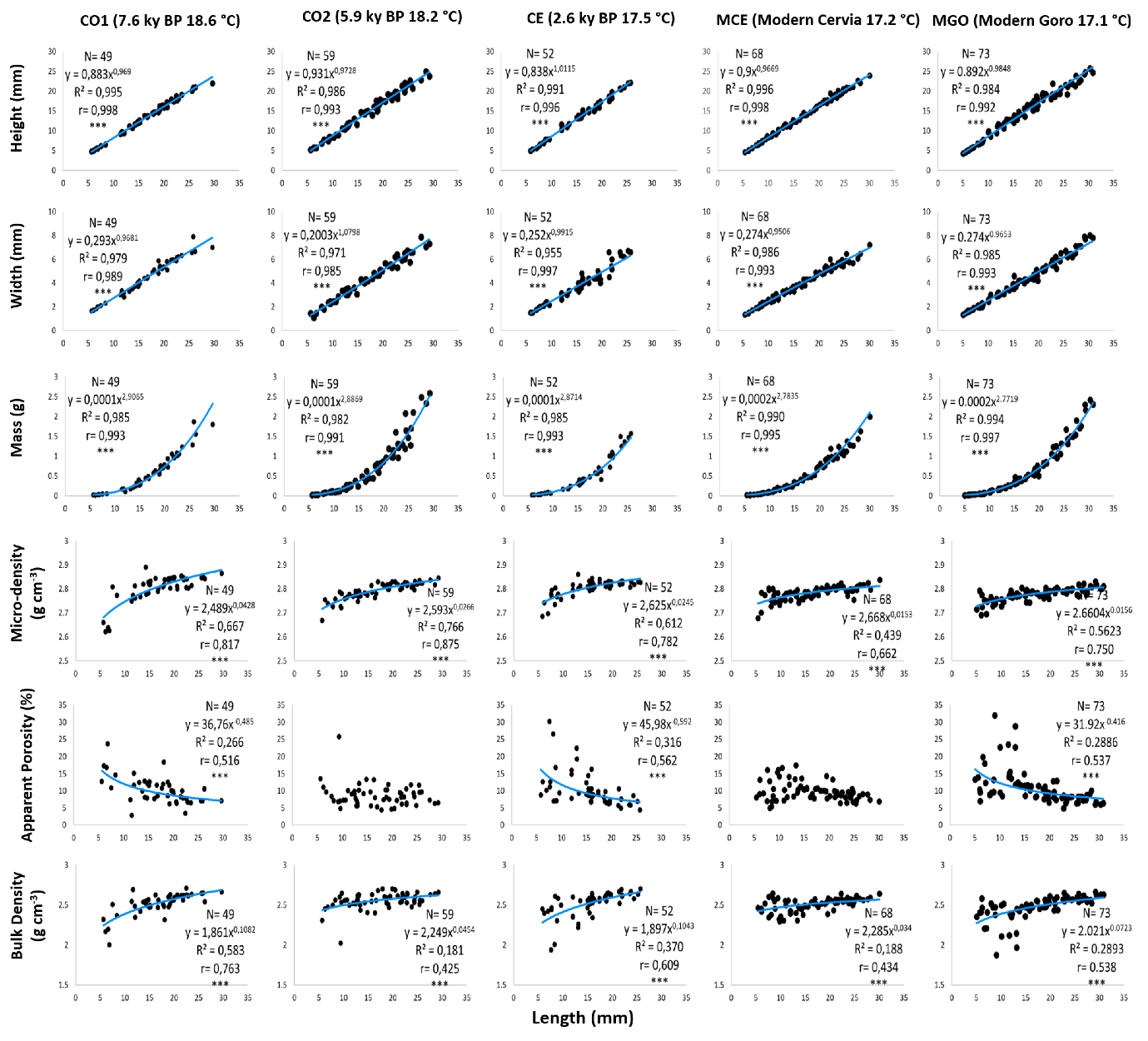
**S1 Fig.** **Correlation of the skeletal and biometric parameters with shell length in the different horizons;** n = number of valves; R^2^ = Pearson’s coefficient of determination*;* r = Pearson correlation coefficient; * p<0.05; ** p<0.01; *** p<0.001.


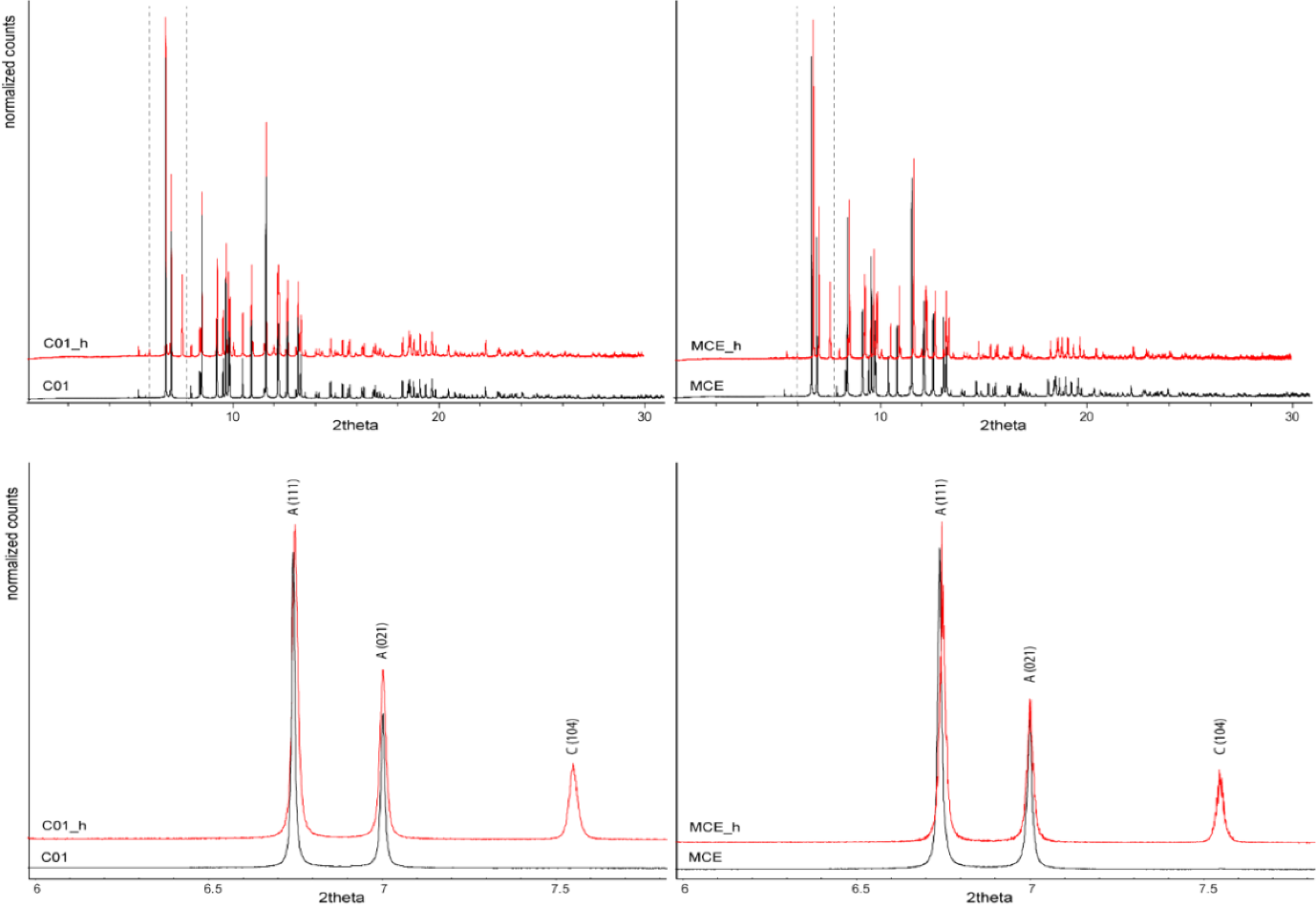
**S2 Fig.** **HR-XRPD patterns of the samples MCE and CO1.** The suffix _h indicates the thermally treated samples. At the bottom enlargements of the diffraction patterns in the 2theta region of main interest are reported. The Miller index is reported among brackets. C = calcite; A = aragonite.


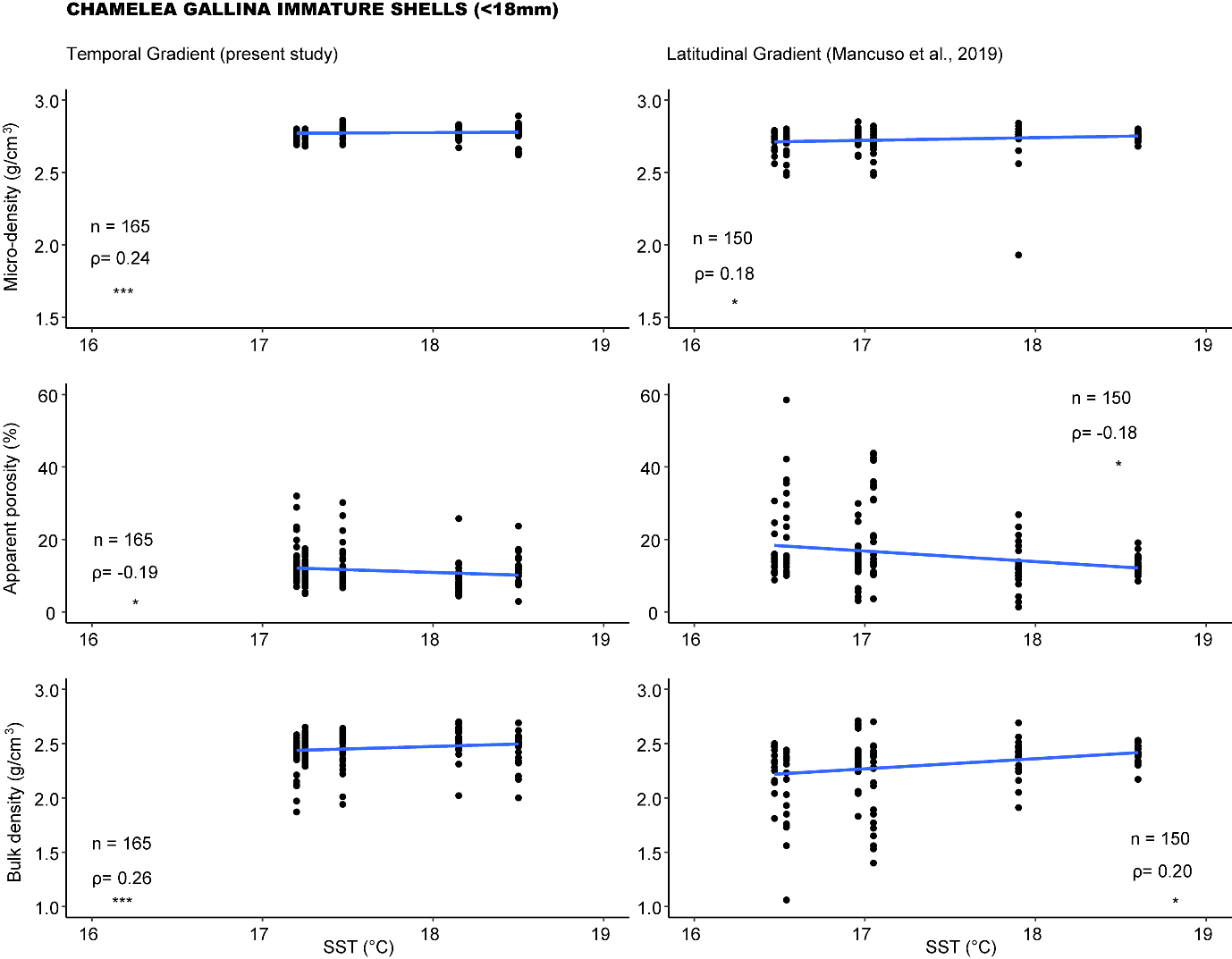
**S3 Fig.** **Comparison of the relationship between shell skeletal parameters and SST in immature shells between this work and Mancuso et al. [11]**; n = number of valves; ρ = Spearman coefficient of determination; * p<0.05; ** p<0.01; *** p<0.001.


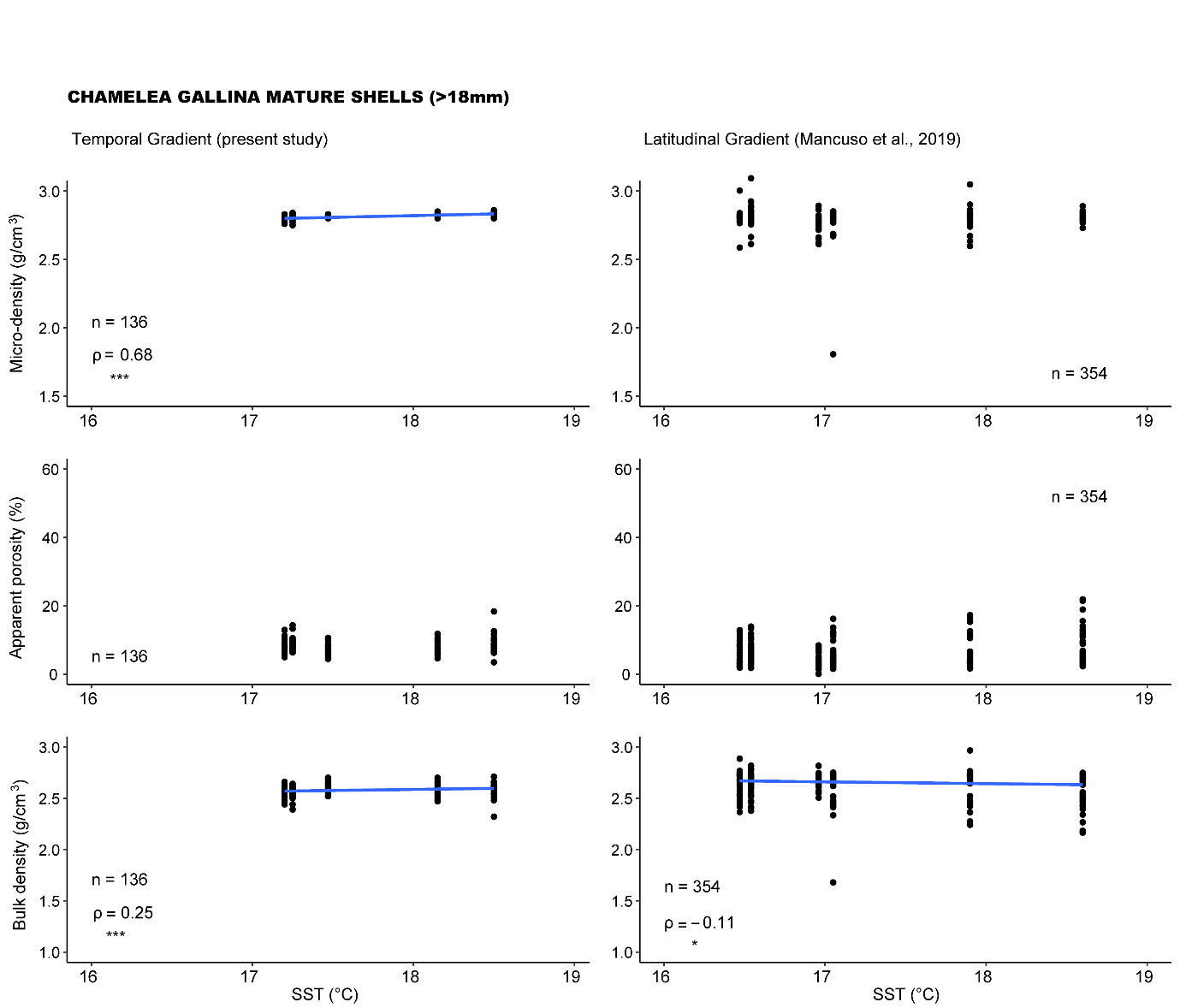
**S4 Fig. Comparison of the relationship between shell skeletal parameters and SST in mature shells between this work and Mancuso et al., [11];** n = number of valves; ρ = Spearman coefficient of determination; * p<0.05; ** p<0.01; *** p<0.001.


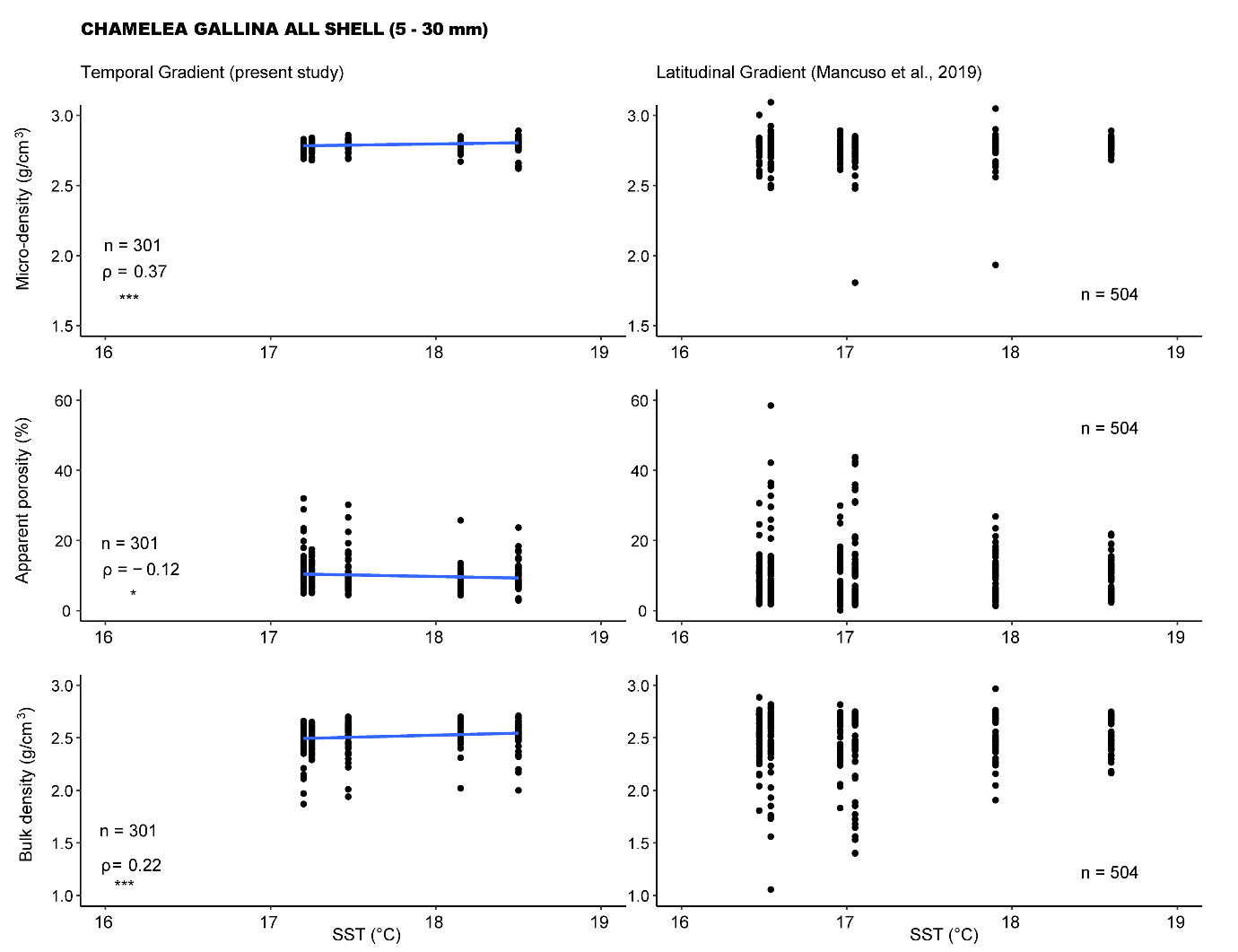


**S5 Fig.** **Comparison of the relationship between shell skeletal parameters and SST for the entire *C. gallina* dataset (i.e., including both immature and mature specimens) between this work and Mancuso et al., [11];** n = number of valves; ρ = Spearman coefficient of determination; * p<0.05; ** p<0.01; *** p<0.001.

| **Horizon** | **^14^C Age** | **SST** | **OM (%)** | **Mg** | **Sr** |
| --- | --- | --- | --- | --- | --- |
|  | **(ky BP)** | **(°C)** |  | **ppm** | **ppm** |
| CO1 | 7.6 ± 0.1 | 18.6 ± 0.4 | 1.72 ± 0.03 | 58 ± 5 | 36 ± 2 |
| CO2 | 5.9 ± 0.1 | 18.2 ± 0.3 | 1.37 ± 0.04 | 60 ± 10 | 40 ± 20 |
| CE | 2.6 ± 0.2 | 17.5 ± 0.5 | 1.60 ± 0.06 | 50 ± 20 | 60 ± 20 |
| MCE | modern | 17.3 ± 0.1 | 1.83 ± 0.02 | 40 ± 10 | 30 ± 9 |
| MGO | modern | 17.2 ± 0.1 | 1.79 ± 0.05 | 50 ± 20 | 40 ± 20 |
| K-W |  |  | * |  |  |

**S1 Table. Intra-skeletal organic matrix and metal content.** Values for each horizon in chronological order. For each parameter mean value and standard error are reported. OM = organic matrix; Mg = Magnesium; Sr = Strontium; K-W = Kruskal-Wallis equality-of-populations rank test; * p<0.05

**Bibliography**

1. Amorosi A, Colalongo ML, Fusco F, Pasini G, Fiorini F. Glacio-eustatic control of continental-shallow marine cyclicity from late quaternary deposits of the southeastern Po Plain, northern Italy. Quat Res. 1999;52: 1–13. doi:10.1006/qres.1999.2049

2. Scarponi D, Kowalewski M. Stratigraphic paleoecology: Bathymetric signatures and sequence overprint of mollusk associations from upper Quaternary sequences of the Po Plain, Italy. Geology. 2004;32: 989–992. doi:10.1130/G20808.1

3. Azzarone M, Pellegrini C, Barbieri G, Rossi V, Gamberi F, Trincardi F, et al. Linking benthic fauna and seismic facies to improve stratigraphic reconstructions: the case of the Mid-Adriatic Deep since the late glacial period (Central Adriatic Sea). Boll della Soc Paleontol Ital. 2020;59: 9–23. doi:10.4435/BSPI.2020.03

4. Bruno L, Campo B, Di Martino A, Hong W, Amorosi A. Peat layer accumulation and post-burial deformation during the mid-late Holocene in the Po coastal plain (Northern Italy). Basin Res. 2019;31: 621–639. doi:10.1111/bre.12339

5. Scarponi D, Azzarone M, Kusnerik K, Amorosi A, Bohacs KM, Drexler TM, et al. Systematic vertical and lateral changes in quality and time resolution of the macrofossil record: Insights from Holocene transgressive deposits, Po coastal plain, Italy. Mar Pet Geol. 2017;87: 128–136. doi:10.1016/j.marpetgeo.2017.03.031

6. Amorosi A, Barbieri G, Bruno L, Campo B, Drexler TM, Hong W, et al. Three‐fold nature of coastal progradation during the Holocene eustatic highstand, Po Plain, Italy – close correspondence of stratal character with distribution patterns. Fielding C, editor. Sedimentology. 2019;66: 3029–3052. doi:10.1111/sed.12621

7. Amorosi A, Bruno L, Campo B, Morelli A, Rossi V, Scarponi D, et al. Global sea-level control on local parasequence architecture from the Holocene record of the Po Plain, Italy. Mar Pet Geol. 2017;87: 99–111. doi:10.1016/j.marpetgeo.2017.01.020

8. Wittmer JM, Dexter TA, Scarponi D, Amorosi A, Kowalewski M. Quantitative bathymetric models for late quaternary transgressive-regressive cycles of the po plain, italy. J Geol. 2014;122: 649–670. doi:10.1086/677901

9. Scarponi D, Angeletti L. Integration of palaeontological patterns in the sequence stratigraphy paradigm: a case study from Holocene deposits of the Po Plain (Italy). GeoActa. 2008;7: 1–13.

10. Maselli V, Trincardi F. Man made deltas. Sci Rep. 2013;3: 1–7. doi:10.1038/srep01926

11. Mancuso A, Stagioni M, Prada F, Scarponi D, Piccinetti C, Goffredo S. Environmental influence on calcification of the bivalve Chamelea gallina along a latitudinal gradient in the Adriatic Sea. Sci. Rep. 2019;9: 11198. doi:10.1038/s41598-019-47538-1
